# Enhancing Membrane Adhesion to Polymeric Substrates via Plasma Treatment

**DOI:** 10.1021/acsapm.5c04051

**Published:** 2026-03-16

**Authors:** Rajan Jain, Christina Carbrello, Kathy Youngbear, Sean Foley, Rong Long, Yifu Ding

**Affiliations:** † Membrane Applications Science, and Technology (MAST) Center, Paul M. Rady Department of Mechanical Engineering, 1877 University of Colorado Boulder, Boulder, Colorado 80309, United States; ‡ MilliporeSigma, 80 Ashby Rd, Bedford, Massachusetts 01730, United States

**Keywords:** adhesion, plasma treatment, membranes, thermoplastics, peel test

## Abstract

Ensuring strong adhesion between porous polymeric membranes and supporting substrates is critical for the reliability and functionality of membrane devices. However, due to the innate low surface energy of polymers, achieving strong chemical bonding between such materials remains challenging. In addition, the small-pore size of membranes often limits effective pore intrusion (necessary for achieving effective mechanical interlocking) by polymer adhesives during high-throughput manufacturing. Plasma treatment is commonly used to modify the surface energy of polymers to improve adhesion and mechanical properties of composite systems. However, it remains unexplored whether the method is effective in improving the adhesion of surfaces containing nanoscale pores as found in membranes. Herein, we demonstrate that adhesion between poly­(ethersulfone) (PES) membranes with 20 and 200 nm pore sizes and polypropylene (PP) substrates is enhanced by low-pressure plasma treatment (power: 30 W, duration: 60 s, gas flow rate: 30 cm^3^/min) of the two surfaces. Thermomechanical bonding between the treated surfaces is performed, and the adhesion behavior is quantified by a T-peel test and imaging analysis. For the 200 nm PES membranes and PP substrate, the adhesion after plasma treatment (152–405 N/m), measured by the interfacial fracture toughness, exhibits an improvement by 0.12 to 2 times in comparison to untreated control samples (114–156 N/m). For the 20 nm PES membranes and PP substrate, the adhesion after plasma treatment (14–242 N/m) exhibits an improvement by 0.13 to 20 times in comparison to that of untreated control samples (12–96 N/m). Among the different types of plasma treatment tested, the oxygen-containing plasmas produce the largest enhancement in adhesion. When benchmarked against the adhesion of densified, nonporous PES film and PP substrates after plasma treatments (0–20 N/m), the adhesion is improved by 13 to 37 times for the 200 nm PES/PP specimens and by 1.5 to 17 times for the 20 nm PES/PP specimens, showcasing the importance of mechanical interlocking due to membrane pore structure for adhesion. This study shows that there is a synergistic effect of chemical bonding and mechanical interlocking on the interfacial fracture toughness between porous membranes and thermoplastic substrates, which can be useful in guiding the membrane bonding process in a variety of applications.

## Graphical abstract

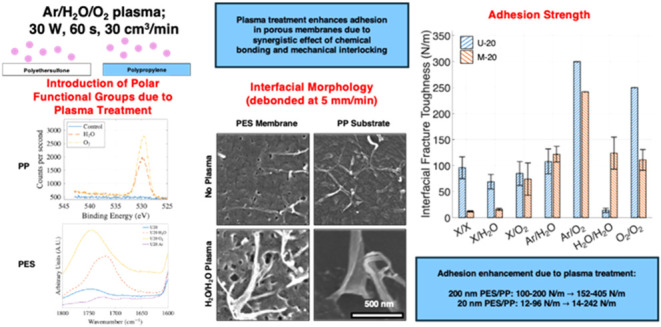

## Introduction

Membrane devices are widely used in microfiltration (MF) and ultrafiltration (UF) applications such as pharmaceutical manufacturing, dairy processing, and bioreactors.^1^ Robust adhesion between the membranes and the supporting substrates is crucial to maintain the integrity of these devices.^2,3^ The lack of proper membrane adhesion can lead to performance issues, such as losing high-value filtrates or allowing potentially detrimental retentates (e.g., pathogens) into the system. Most membrane processes utilize pressure to drive the feed through the system, and it is important for the devices to withstand such mechanical loading during operation.^4–6^

Membranes are adhered to substrates by either thermal welding^7^ or resin curing^8^ with high-throughput processes. In general, chemical interactions between polymeric substrates (e.g., polypropylene or PP) and membranes (e.g., PVDF or PES) are weak. To achieve strong adhesion, mechanical interlocking is necessary, which requires the effective pore intrusion of the substrate polymer. For the resin-curing process (e.g., epoxy-based or urethane-based adhesives), this problem is not encountered because the low-viscosity precursors can quickly infiltrate the pores prior to curing. However, these adhesives can be unstable under γ irradiation,^9^ which is often used to sterilize membrane devices.^10,11^

On the other hand, it can be challenging for pore intrusion by thermoplastic substrates (e.g., PP) under high-throughput thermal welding processes^12^ due to three fundamental limitations of materials/membrane properties. First, the high viscosity of the infiltrating polymer at the bonding temperature may impede the pore intrusion process. Although the viscosity of polymers can be reduced by either reducing the molecular weight or increasing the bonding temperature, the former would result in significantly lowering toughness and strength of the polymer,^13^ and the latter is limited by the glass transition temperature (*T*_g_) or melting temperature (*T*_m_) of the membrane. Second, small pore sizes of the MF or UF membranes imply slow pore intrusion kinetics.^14^ Last, compressibility of the porous membrane can lead to pore collapse under increasing pressure (the driving force for pore intrusion), which in turn limits the extent of pore intrusion.^15,16^ From our recent study^12^ of thermomechanical bonding between PES membranes and PP substrates, increasing the bonding pressure from 1 to 7 MPa significantly reduced the adhesion strength for 20 nm PES membranes. Specifically, the peel strength for 20 nm PES membranes bonded with high molecular weight PP was as low as 9 N/m,^12^ which is insufficient for practical applications.

Plasma treatment of low surface energy materials^17^ like polymers has been commonly used for improving wettability or surface energy,^18–26^ which can lead to adhesion enhancement.^27–30^ For membrane applications, plasma treatment has been applied to modify the membrane surface to improve process efficiency.^29,31^ For example, corona discharge has been used to finetune the pore size distribution in membranes cast by phase inversion.^32^ Fine-tuning the surface roughness of poly­(dimethylsiloxane) (PDMS) membranes was demonstrated by controlling the electron density, temperatures of noble gases in the plasma, and molecular weight of the PDMS.^33,34^ Surface roughness enhancement has effectively shown enhanced adhesion between graphene membranes and silicon dioxide substrates.^35^ Plasmas made with noble gas admixtures with oxygen have been used to decrease the water contact angle of PES membranes^36^ and medical plastics.^37^

In this study, we systematically investigate the use of a low-pressure plasma treatment to enhance adhesion between PES membranes and PP substrates. Specimens of PES membranes and PP substrates were treated with different plasmas, including Ar, H_2_O, and O_2_, and then thermomechanically bonded. The adhesion behaviors of PES/PP with different combinations of plasma treatment (PT) were systematically determined using T-peel tests and imaging analysis. The results reveal that PT can significantly improve the membrane adhesion strength. For example, the O_2_ PT improved the adhesion strength of 20 nm PES membranes by up to 20 times, which makes it an attractive method to overcome the limitations of conventional thermomechanical bonding.

## Materials and Methods

This study uses the same membranes as those in our previous study:^12^ asymmetrical PES membranes of two pore sizes (20 and 200 nm) manufactured by MilliporeSigma using the standard nonsolvent-induced phase separation method. For each pore size rating, two different membrane chemistries were used: unmodified (U) and acrylamide-modified (M). For the sake of convenience, the two 20 nm PES membranes will be referred to as U-20 and M-20, while the two 200 nm PES membranes will be referred to as U-200 and M-200. Table 1 summarizes the mechanical and pore structure characteristics of the unmodified membranes, where *E* and σ_*y*_ are the elastic modulus and yield strength at 180 °C (the bonding temperature), and Φ and Φ_*s*_ are the overall and surface porosities, respectively. Note that chemical modification does not significantly change the physical and mechanical properties of the membranes. The *T*_g_ values of the membranes are determined to be about 220 °C by dynamic mechanical analysis.

**Table 1:** Properties of Unmodified PES Membranes and PP Substrate

membrane	thickness (μm)	*E* (MPa)	σ_*y*_ (MPa)	Φ (%)	Φ_*s*_ (%)	nominal pore diameter (nm)^a^
U-200	180	58.1	1.7	80	18	200
U-20	140	67.9	3.0	73	10	20

polymer	*M*_*n*_ (g/mol)	PDI	η_0_ (Pa·s)	*T*_m_ (°C)	*f*_c_ (%)	*T* (MPa)
PP	114,000	1.83	1650	154.2	58	92.8

a Based on the manufacturer’s rating.

PP is commonly used as a supporting material for membrane devices due to its chemical stability, biocompatibility, and low manufacturing cost. Relevant properties of the PP film used in this study are summarized in Table 1, where *M*_*n*_ is the number-average molecular weight, PDI is the polydispersity index, η_0_ is the steady-state viscosity at 180 °C (the bonding temperature used in this study), *f*_c_ is the degree of crystallinity, and *T* is the tensile toughness at room temperature, respectively.

### Plasma Treatment and Characterization

Plasma treatments of PES membranes (the active or contacting side, where pore size is either 20 or 200 nm, respectively) and PP were conducted in a plasma cleaner (PIE Scientific, Tergeo Plus). This instrument generates plasma in RF mode at 13.56 MHz. Water vapor (H_2_O), oxygen (O_2_), and argon (Ar) gases were used as process gases to generate continuous plasma (at 30 cm^3^/min, 30 W) inside the specimen chamber. To simplify the nomenclature of plasma-treated PES/PP specimens, an “A/B” PT condition refers to one with the PES surface treated with “A” plasma and the PP surface treated with “B” plasma. “X” refers to no PT of the respective surface. Six PT conditions, namely, Ar/H_2_O, Ar/O_2_, H_2_O/H_2_O, O_2_/O_2_, X/H_2_O, and X/O_2_, were investigated to compare the effects of different reacting species in the plasma on the adhesion between the PES and PP surfaces. Note that the Ar PT on PP was not examined because the neutral plasma species would not lead to an increase in polarity of the PP. For every PT condition, six replicate samples were treated, each measuring 13 mm × 44 mm. The experiments described above were carried out for all four membranes: U-20, M-20, U-200, and M-200.

The roughness of plasma-treated surfaces was quantified with an atomic force microscope (DriveAFM, Nanosurf). Surface energies of the plasma-treated samples were determined using a two-liquid wetting method:^38^ by determining the contact angles of water (polar probing liquid) and diiodomethane (nonpolar probing liquid) droplets (2 μL) on the plasma-treated surfaces. To remove the impact of pores on the contact angle measurements, dense PES films were prepared by densifying U-20 and M-20 under 275 °C and a pressure of 4.3 MPa for 5 min. The permeance of U-20 and M-20 membranes after different PT was measured using DI water permeation experiments with a dead-end filtration cell (Sterlitech, HP4750) with an active membrane area of 11.5 cm^2^. The membranes were prewetted by soaking in a 50:50 isopropanol/DI water solution for 5 min. The permeate mass was recorded every minute using an automated electronic balance. All the permeation experiments were conducted at room temperature and 20 psi (138 kPa) gauge pressure. For each 20 nm PES membrane, three measurements were carried out and average permeance values are reported. Scanning electron microscopy (Hitachi SU3500 VP SEM and Hitachi SU8600 FESEM) was used to image surfaces of the PES after plasma treatment. For each sample, a 2 nm layer of platinum was deposited to reduce the charging effect.

### Thermomechanical Bonding of Membranes and PP

The thermomechanical bonding process was carried out within 1 h of PT using a nanoimprinter (NIL, Eitre 3, Obducat), which offers precise temperature and pressure control. The plasma-treated surfaces of the PES and PP were aligned in contact with a Kapton film (thickness = 50.8 μm) separating them to create noncontact areas between the two surfaces to allow for peel testing, similar to the previous work.^12^ All samples were bonded at 180 °C and 1 MPa for 15 s. A 3M Scotch tape was attached to the membrane side of the sample to reinforce it for the following peel test.

### Adhesion Measurements

A modified T-peel test based on ASTM D3330 ^39^ was performed using a UTM (5965, Instron equipped with a 50 N load cell) to determine the adhesion strength of the bonded specimens. For a given sample, the noncontact areas were mounted in the upper and lower grips of the UTM, and a small amount of prestrain was applied to straighten the sample. The upper grip was raised at 5 mm/min, corresponding to a strain rate of approximately 15%/min until the sample either fractured or debonded completely. For each PT condition, six samples were tested, and the average values of the interfacial fracture toughness with corresponding standard deviations are reported. SEM was used to image the surfaces of PES and PP after debonding. In addition, the debonded surfaces were imaged with AFM using a HQ:NSC15/Al BS probe (8 nm tip diameter).

## Results

### Properties of the Membranes and PP after Plasma Treatments

The surface energy of the plasma-treated PES and PP surfaces was measured by analyzing the contact angles (θ) of water (DIW; a polar probing liquid) and diiodomethane (DIM; a nonpolar probing liquid); the contact angles of DIW and DIM on both materials are shown in Figure S1. The surface energies (γ_lg_) with dispersive (γ_lg_^d^) and polar (γ_lg_^p^) components of both liquids are summarized in Table S1.^38^ Based on these values and the contact angles of the two liquids, the Owens–Wendt–Rabel–Kaelble equation was used to calculate the surface energy of the samples

1 $$\gamma_{\mathrm{lg}}\left(1+\cos\left(\theta \right)\right)=\frac{4\gamma_{\mathrm{sg}}^{d}\gamma_{\mathrm{lg}}^{d}}{\gamma_{\mathrm{sg}}^{d}+\gamma_{\mathrm{lg}}^{d}}+\frac{4\gamma_{\mathrm{sg}}^{p}\gamma_{\mathrm{lg}}^{p}}{\gamma_{\mathrm{sg}}^{p}+\gamma_{\mathrm{lg}}^{p}}$$

The second term in eq 1 becomes zero when solving diiodomethane contact angles, and solving for the two components γ_sg_ yields the surface energy of the treated surface. The surface energy values of the plasma-treated PP surfaces are summarized in Figure 1b. The surface energy of PP after O_2_ PT is comparable to a literature report under similar power and duration.^38^ XPS scans from the same study show an increase in the oxygen content on the PP surface, forming a range of polar bonds.^38,40^ Chemical changes due to PT were observed by XPS and FTIR spectroscopies for PP (Figure 1a) and PES, respectively. Figure S2 shows a comparison of the C 1s and O 1s spectra of PP treated with H_2_O and O_2_ PT with Gaussian fits to estimate the amount of C–O and C=O bonds. Note: The O 1s spectrum of control PP contains only noise because it does not have any oxygen-containing functional groups. It is evident that the amount of C=O character is highest for the O_2_ plasma. On the other hand, Figure S3 compares the FTIR spectra in two regions: 1600–1800 cm^–1^ and 3000–3500 cm^–1^ of all membranes used in this study. For M-20 and M-200, as seen in Figure S3­(a,b): a peak between 3300 and 3400 cm^–1^ appears due to an overlap of the hydroxyl group and N–H stretching; a peak between 1650 and 1680 cm^–1^ due to amide C=O stretch (absent for U-20 and U-200); and a peak with a shoulder between 1700 and 1750 cm^–1^ due to a combination of carbonyl groups, amide stretching, and hydrogen-bonding from the polar groups. For U-20 and U-200, as seen in Figure S3­(c,d): there is a weak, broad peak between 3300 and 3400 cm^–1^ from adsorbed hydroxyl groups; and a peak between 1700 and 1780 cm^–1^ due to carbonyl groups. In all samples, carbonyl groups are incorporated due to plasma. M-20 and M-200 have N–H containing groups and hence a more polar nature than those of U-20 and U-200.

**Figure 1:**
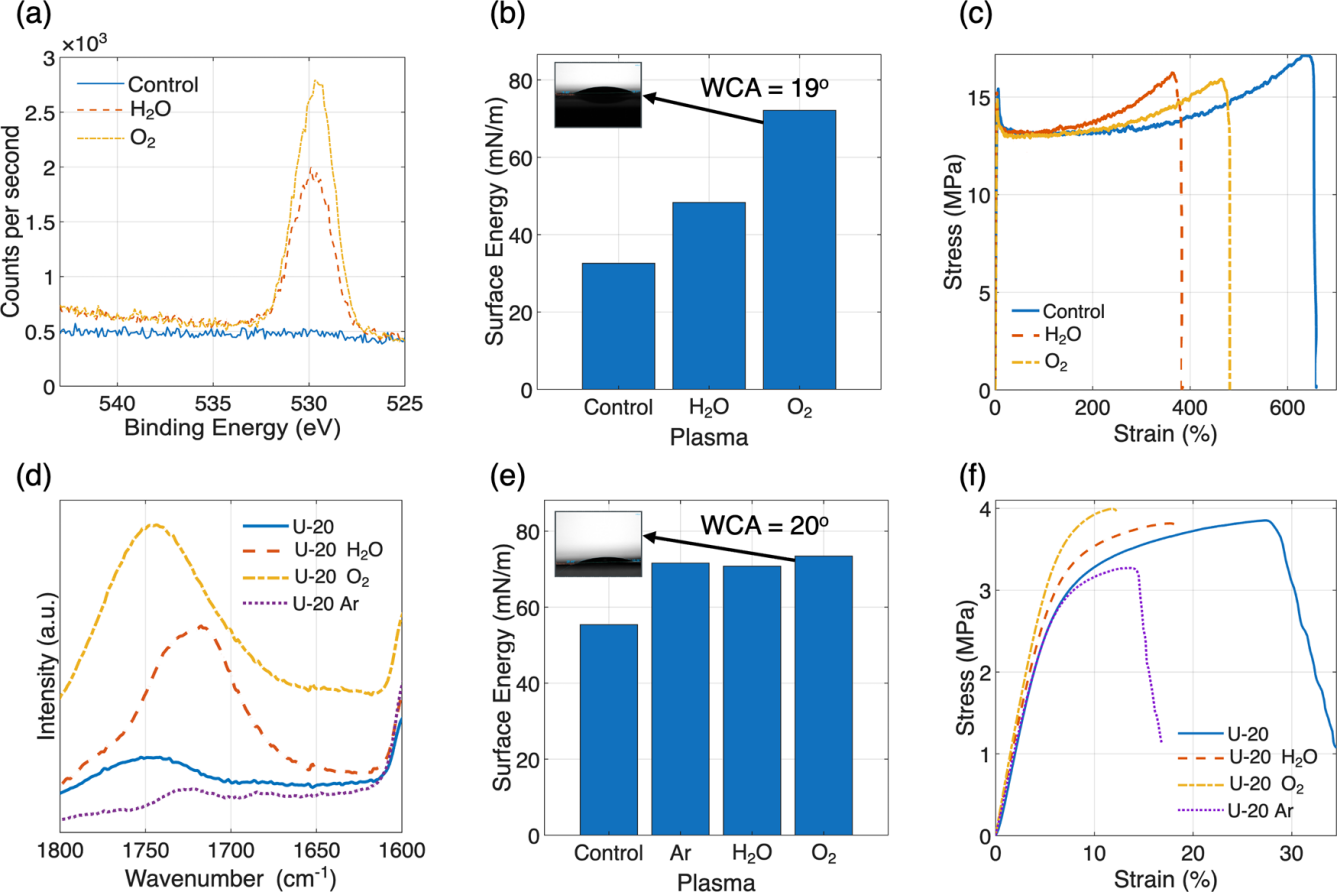
(a–c) XPS O 1s spectra, surface energy, and uniaxial tensile response of PP subjected to H_2_O and O_2_ plasmas. (d–f) FTIR spectra (1600–1800 cm^–1^), surface energy, and uniaxial tensile response of U-20 subjected to Ar, H_2_O, and O_2_ plasmas.

The stress–strain curves (Figure 1c) suggest that the PT had negligible impacts on the elastic response and mechanical strength of the PP films. This is reasonable considering the chemical changes resulting from PT are localized on the surface of the films. Carbon fiber-polyamide-11 composites treated with atmospheric plasma showed 10 times increase in the lifetime compared with untreated composites for similar stress loading.^41^ Similarly, cyclic loading (∼10^4^ cycles) on atmospheric plasma-treated nylon-66 plates bonded with polyurethane-based adhesives showed that PT was more effective than abrasion-based pretreatment for resisting failure.^42^ However, the PT reduced the elongation-at-break, suggesting that the surface chemical modifications^43^ increased the probability of crack initiation at the surface. Note that such embrittlement is common for surface treatments, including plasma-treated polymers.^44,45^ Nevertheless, the PT-treated PP films still possess excellent strength (16–17 MPa), ductility (failure strain >350%), and toughness (tensile toughness >53 MPa) for practical applications. Plasma treatment has been used to improve fracture toughness by promoting fiber bridging and breakage instead of fiber pullout in carbon fiber/PEKK composite joints^46–48^ and nylon-66,^49^ and increased modulus and toughness of aramid/epoxy composites.^50,51^

From AFM measurements as seen in Figure S4, the RMS roughness of the PP surface decreases slightly, from 25.4 nm (untreated surface) to 19.8 nm (for H_2_O plasma) and 21.2 nm (for O_2_ plasma), which is consistent with a literature report^52^ showing that PP experiences surface smoothening for short PT durations while roughening thereafter. The similar surface roughness for all of the PP samples confirms that the increase in surface energy (calculated from the contact angles) after PT is directly caused by the chemical changes, specifically, the increase of polar content.

Similar characterizations were carried out for the PES membranes after different PT. Using the U-20 membrane as an example, Figure 1e,1f show the surface energy and stress–strain response, respectively. Note that the surface energy measurements were conducted on densified PES membranes to eliminate the impact of pores on the contact angles of the wetting liquids. Like PP films, PES films display an increase in surface energy upon PTs. A similar trend was observed for PES films densified from M-20 membranes (Figure S5a).

Note that the variation in yield strength (onset of the stress plateau) for different samples was most likely attributed to the variation of the untreated membrane samples. Such variation was less evident for M-20 membranes (Figure S5b). Figure S6 shows the topography of the U-20 and M-20 membranes before and after treatment with O_2_ plasma, while the same for U-200 and M-200 membranes is shown in Figure S7. The RMS roughness of the U-20 membrane increased from 4.7 to 39 nm after the O_2_ PT. In comparison, the RMS roughness of the M-20 membrane only slightly increased from 2.7 to 3.4 nm. A similar trend was observed between U-200 and M-200: RMS increased from 58 to 135 nm for U-200 and from 58 to 71 nm for M-200, respectively. Note that the RMS roughness in unmodified membranes is attributed to the presence of pores. The data suggests the acrylamide coating can alleviate the roughening under current PT conditions.

Figure 2(a) shows the surface of the U-20 and M-20 membranes before and after the O_2_ PT. It is evident that PT causes the membrane surface to be etched away due to the oxidative action of different plasma species. However, the U-20 shows more etching than the M-20 membrane. The surfaces of both membranes after treatments with Ar and H_2_O plasmas are shown in Figure S8. Figure 2(b) shows the permeance of the U-20 and M-20 membranes after different PT. As expected, the permeance of the membranes with the different PT increases from the baseline permeance without PT. However, it must be noted that this does not affect the membrane permeance in devices, as the PT is performed only in the impermeable bonding area.

**Figure 2:**
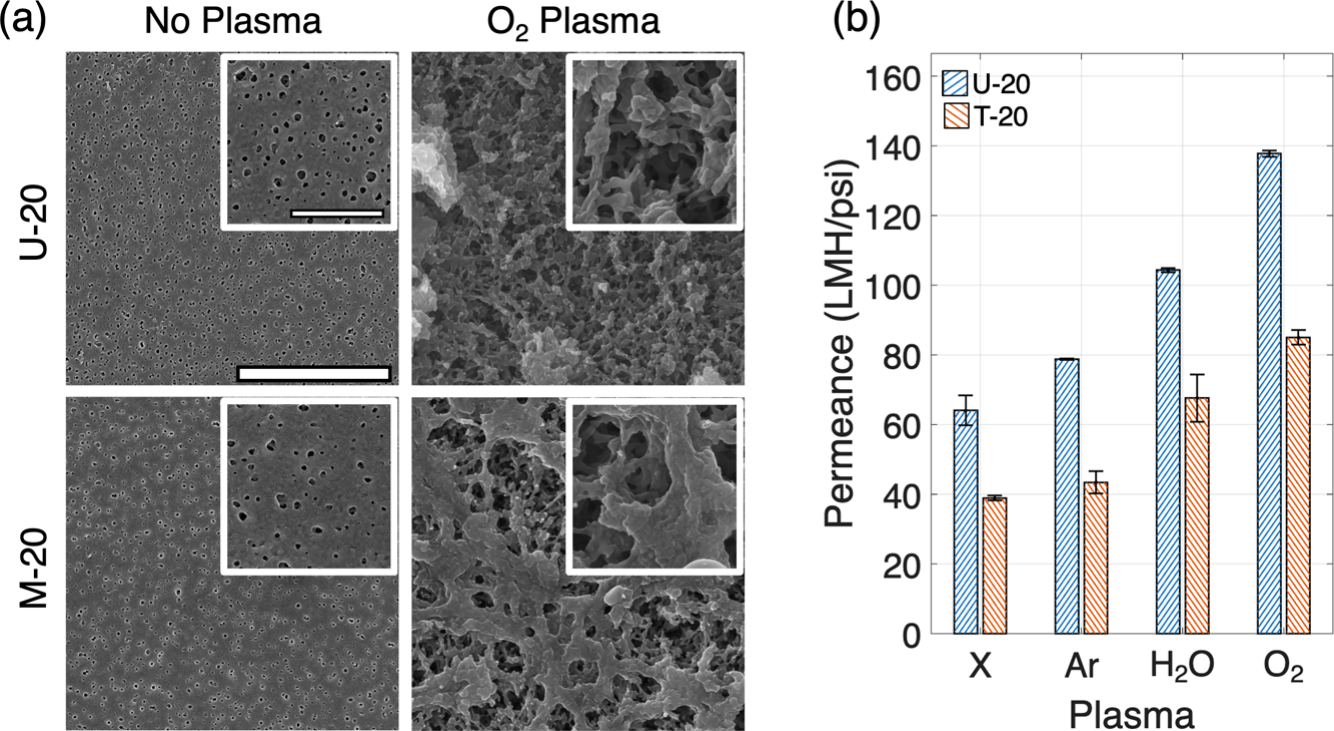
(a) Membrane surfaces of U-20 and M-20 without PT and with O_2_ PT (scale bar is 5 μm and inset scale bar is 500 nm), and (b) permeance of U-20 and M-20 membranes after different PT.

### Adhesion between Dense PES Films and PP

To assess the contributions of mechanical interlocking and chemical interactions, the adhesion strength between dense PES films with PP, after different PTs, was first determined. Particularly, the interfacial fracture toughness, *G*_c_, was determined from the peel tests through the following equation

2 $$G_{c}=\frac{2F_{peel}}{w}$$

where *F*_peel_ was the steady-state peel force obtained from force–displacement curves of the peel test (Figure 3), and *w* is the width of the bonded sample.

**Figure 3:**
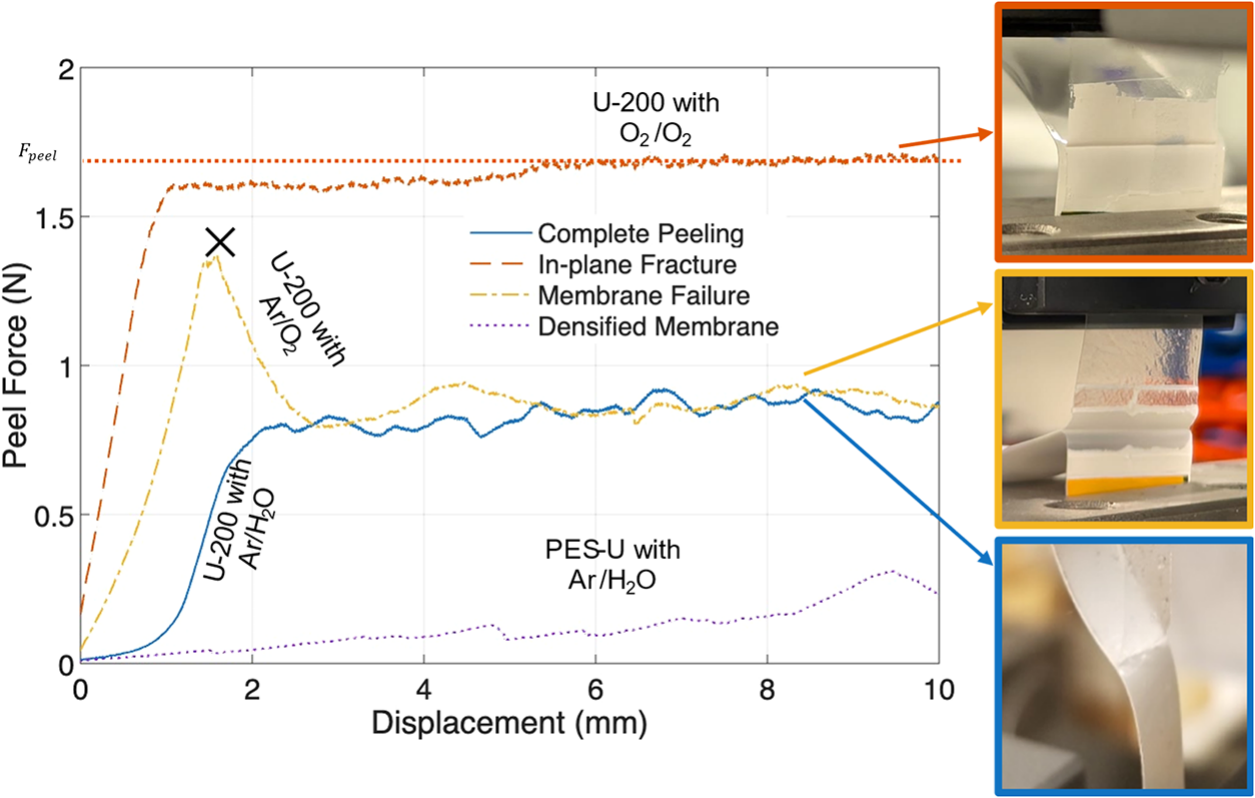
Comparison of typical peel behavior in PES/PP specimens showing different failure mechanisms: interfacial failure (blue and purple), membrane failure (yellow), and in-plane fracture (red) seen in PES/PP specimens after different PT. “X” on the yellow curve denotes the occurrence of membrane failure.

Figure 4a summarizes the interfacial fracture toughness for PES films densified from both U-20 (labeled as PES-U) and M-20 (labeled as PES-M). Without PT, PES films (PES-U and PES-M) and PP did not bond to each other, which is consistent with the poor thermodynamic adhesion between PES and PP (about 26 mN/m).^53^ For PES-U samples, treating PP alone did not substantially improve the adhesion strength. Modest adhesion strengths (5–20 N/m) were observed when PES-U and PP were both treated. The adhesion strength appears to correlate well with the surface energy of the plasma-treated PES-U and PP (Figure 1): with O_2_/O_2_ treatment resulting in the highest surface energies for both surfaces, and correspondingly, the largest adhesion strength. Such correlation is also observed in PES-M samples (Figure S5a), with the only exception of X/O_2_. From the SEM images, the debonded surfaces of both PES and PP generally appear featureless (Figure 4b). Such morphologies are consistent with the weak adhesion seen on these specimens. Isolated small patches of fractured polymers were observed for PT samples, as shown in the example of X/H_2_O, which is most likely caused by limited mechanical interlocking resulting from the local roughness and defects on the PES films.

**Figure 4:**
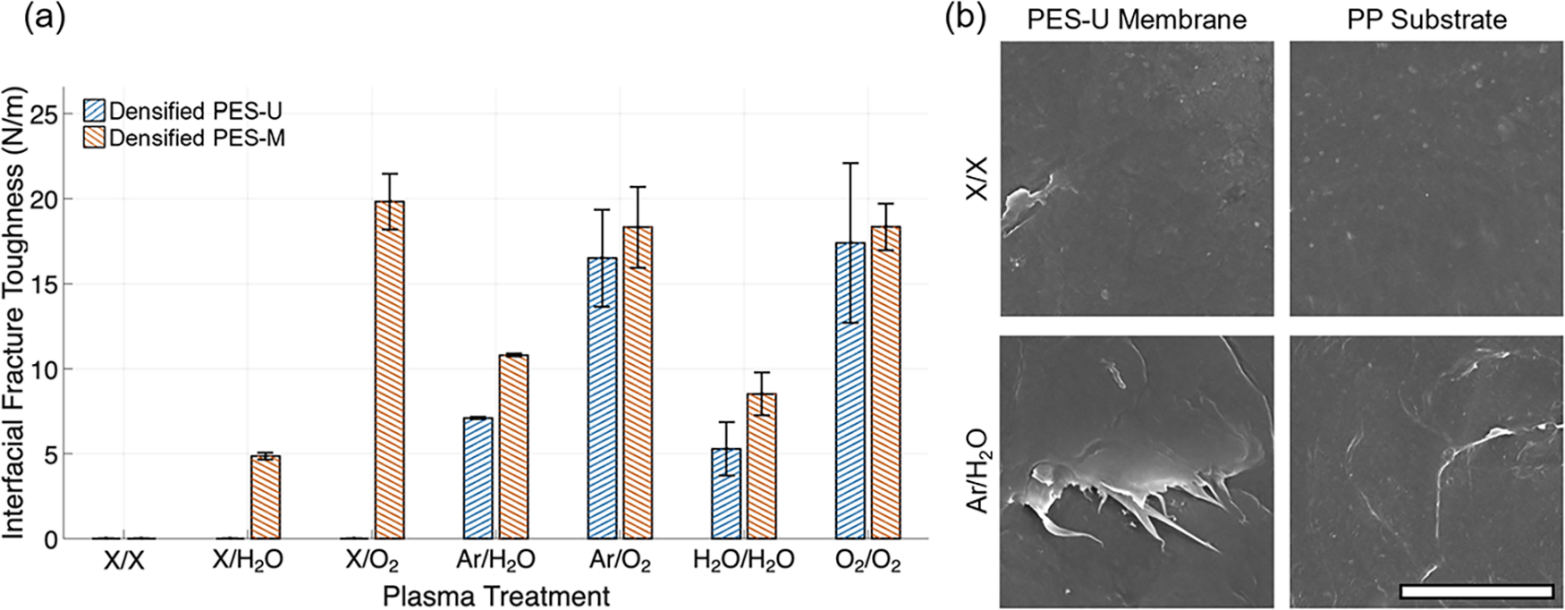
(a) Comparison of interfacial fracture toughness between PES films (PES-U, densified from U-20, and PES-M, densified from M-20) and PP after different PT. (b) Representative SEM images of the PES-U (left column) and PP (right column) after debonding from no PT (X/X) and from Ar/H_2_O treatment.

### Adhesion between 200 nm PES Membranes and PP

In a previous work,^12^ it was demonstrated that the dominant debonding mechanism was interfacial failure for thermomechanically bonded PES/PP. The representative curve for the same is very similar to U-200/PP with Ar/H_2_O shown in Figure 3. Correspondingly, the *G*_c_ value was dictated by the pullout and fracture of the infiltrated PP fibers. For 200 nm PES/PP specimens without PT, *G*_c_ values ranged between 100 and 200 N/m, depending on the specific bonding pressure and membrane chemistry.

As summarized in Figure 5a, plasma treatment successfully enhanced the *G*_*c*_ values for the 200 nm PES membranes by about 10–220%. No major differences were observed between U-200 and M-200 in terms of the effects of PT. Interfacial fracture remains the dominant mechanism for most samples. However, membrane failure during debonding was also observed when the adhesion strength reached above the fracture strength of the membranes. Specifically, membrane fracture through the thickness was observed for the U-200 membrane with O_2_/O_2_ PT (shown in Figure 3) and M-200/PP with Ar/O_2_ PT. In the case of U-200/PP treated with Ar/O_2_, in-plane fracture was observed during debonding (Figure 3), where the fracture occurred by splitting the U-200 membrane. Overall, the O_2_ plasma-treated PP showed consistent improvement in *G*_c_ values when bonded with the 200 nm PES membranes regardless of membrane chemistry and plasma treatment. In comparison, water vapor plasma results in a low to moderate increase in *G*_c_ of the 200 nm membranes. In general, the data are consistent with the largest increase in surface energy of PP (Figure 1) and the corresponding trend of *G*_c_ for dense PES/PP (Figure 4a).

**Figure 5:**
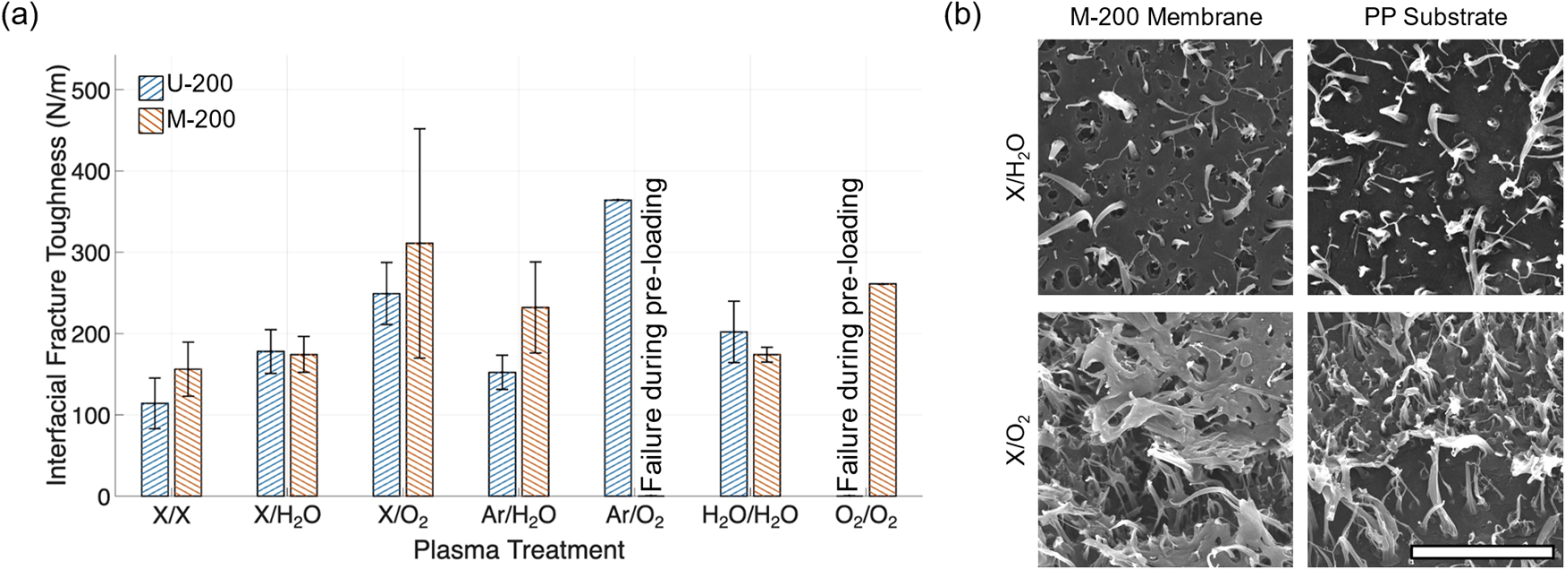
(a) Comparison of interfacial fracture toughness of 200 nm PES/PP specimens subjected to different PT, with sample failure due to preloading observed in M-200 (Ar/O_2_) and U-200 (O_2_/O_2_). Note that only one sample in the Ar/O_2_-treated U-200 was successfully tested, while other samples were fractured during handling, which is the reason for the missing error bars for this system. (b) Representative SEM images of the M-200 (left column) and PP (right column) after debonding from no PT (X/X) and from X/O_2_ treatment. The scale bar is 5 μm.

The ratio of *G*_c_ between PP bonded with 200 nm PES membranes (data in Figure 5a) and PP bonded with dense PES films (data in Figure 4a) ranges between 1300 and 3700% (as seen in Figure S9, neglecting the U-200/PP with Ar/O_2_, and M-200/PP with O_2_/O_2_). The ratio is significantly higher than the ratio between samples treated with and without plasma (10–220%), which highlights the dominant role of mechanical interlocking in improving the adhesion strength of the 200 nm membranes. Figure 5b shows the representative fractography of the debonded PES and PP surfaces to emphasize the difference between the interfacial failure (M-200 with the X/H_2_O PT) and the in-plane fracture (M-200 with the X/O_2_ PT) debonding modes. In typical interfacial failure, elongation and fracture of infiltrated PP nanofibers were evident, while no clear deformation or damage to the PES membranes was observed. In contrast, in-plane membrane fracture shows the damaged, partially broken PES membrane surface left on both surfaces, in addition to the fracture of infiltrated PP nanofibers. The in-plane fracture observed in Figure 3 apparently occurred near the top surface of the PES membrane. Nevertheless, the additional energy associated with PES membrane deformation further enhanced the interfacial fracture toughness.

As we reported recently,^12^ the *G*_c_ can be estimated by summing the fracture energy of all the infiltrated PP fibers

3 $$G_{c}=\rho_{f}\frac{\pi d_{f}^{2}}{4}L_{0}T$$

where ρ_f_, *d*_f_, *L*_0_, and *T* are the average diameter, areal density, infiltration depth, and tensile toughness of the PP fibers. Values of *L*_0_ were estimated from the capillary infiltration kinetics and Darcy’s law, while *T* was determined by uniaxial tensile testing.^12^ The values of ρ_f_ and *d*_f_ were estimated from the SEM images. Table 2 summarizes the estimated *G*_*c*_ based on the PP fibers observed on the debonded 200 nm PES/PP surfaces. Note that data are absent for M-200/PP with X/O_2_ (membrane failure), and the PP surface of U-200/PP with H_2_O/H_2_O (no fibers observed on the surface). The *G*_c_ values estimated from eq 3 appear to be slightly lower than the experimentally determined *G*_c_ value (Figure 5). The small discrepancy could be attributed to the fact that eq 3 focuses on the energy required to stretch and fail the PP fibers and does not account for the enhanced chemical interactions between PES and PP due to plasma treatment.

**Table 2:** PP Fibers on the Debonded Surfaces of the 200 and 20 nm PES/PP Specimens after Different PTs

		X/H_2_O		X/O_2_		Ar/H_2_O		H_2_O/H_2_O	
sample	property	PES	PP	PES	PP	PES	PP	PES	PP
U-200	*d*_f_ (nm)	347 ± 156	310 ± 52	486 ± 62	420 ± 120	308 ± 46	364 ± 84	311 ± 186	
	ρ_f_ (fibers/μm^2^)	1.37	1.43	0.98	0.68	0.59	0.78	0.59	
	*G*_c,est_ (N/m)	191	159	268	139	65	120	66	
M-200	*d*_f_ (nm)	229 ± 56	258 ± 58			302 ± 109	355 ± 45	420 ± 128	340 ± 82
	ρ_f_ (fibers/μm^2^)	1.52	1.18			1.80	1.00	1.19	1.13
	*G*_c,est_ (N/m)	92	91			188	146	243	151
U-20	*d*_f_ (nm)	23 ± 7	26 ± 12	23 ± 3	25 ± 5	36 ± 12	33 ± 11	40 ± 9	30 ± 6
	ρ_f_ (fibers/μm^2^)	9.7	48.0	27.4	48.2	53.4	32.5	11.9	20.4
	*G*_c,est_ (N/m)	2	11	5	10	23	12	6	6
M-20	*d*_f_ (nm)	25 ± 13	21 ± 5	24 ± 5	21 ± 4		33 ± 45	23 ± 11	48 ± 8
	ρ_f_ (fibers/μm^2^)	12.7	23.9	17.3	29.9		26.4	10.0	23.9
	*G*_c,est_ (N/m)	3	3	3	4		9	2	18

### Adhesion between 20 nm PES Membranes and PP

Figure 6a summarizes the *G*_c_ values for the 20 nm membranes after different PTs. Compared with the 200 nm membranes, significant differences were observed between the two membrane chemistries. For U-20 membranes, significant increases (∼200% and ∼300%) in *G*_c_ were observed only for Ar/O_2_ and O_2_/O_2_ plasmas. Strangely, samples treated with H_2_O/H_2_O plasma showed a significant reduction. In contrast, all PT increased the *G*_c_ values for M-20 membranes from 12 N/m for untreated samples (X/X) to ∼240 N/m for samples treated with Ar/O_2_, whose debonded surfaces are shown in Figure 6b.

**Figure 6:**
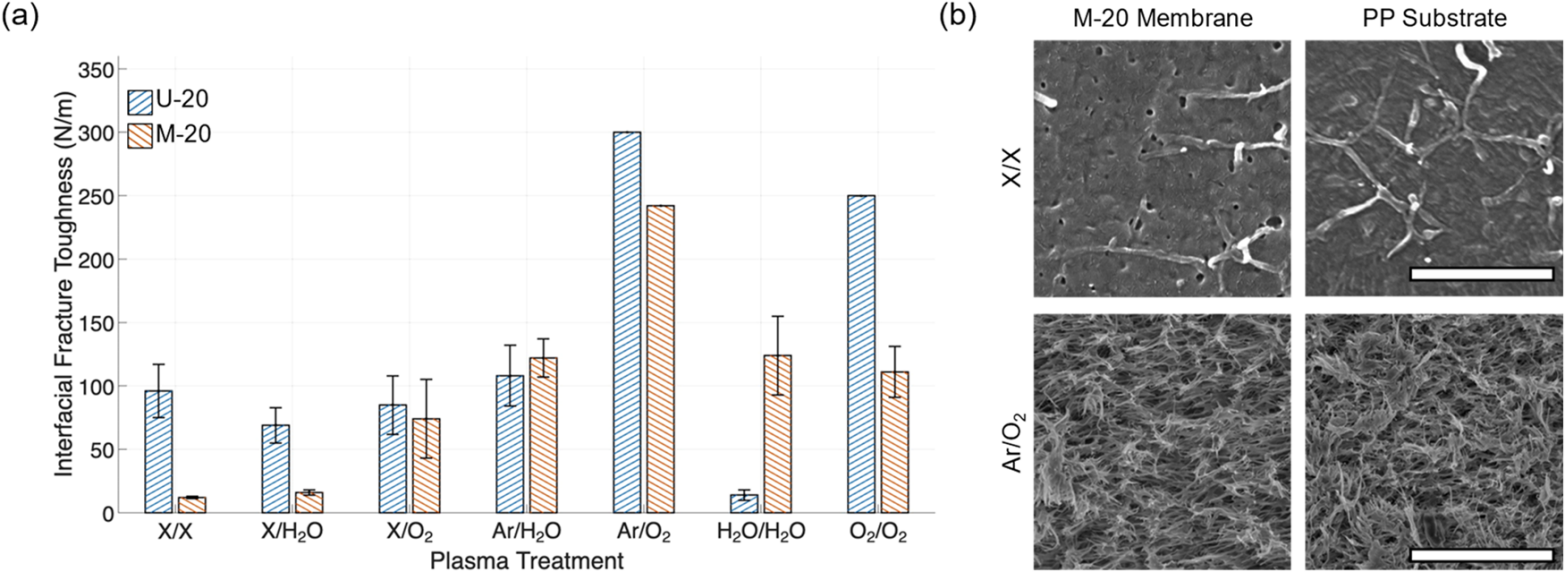
(a) Comparison of interfacial fracture toughness of 20 nm PES/PP specimens subjected to different PT. (b) Representative SEM images of the M-20 (left column) and PP (right column) after debonding from no PT (X/X; scale bar is 500 nm) and from Ar/O_2_ treatment (scale bar is 5 μm).

The relative enhancement of *G*_c_ in porous 20 nm membranes with respect to dense PES films is plotted in Figure S10. The porous nature of the membranes contributes to an increase of approximately 150–1700% in *G*_c_ as compared to nonporous, dense PES films. This ratio is within the same range as the 200 nm membranes discussed above, again highlighting the important role of interlocking with the infiltrated PP. Figure 7 shows representative SEM images of the surfaces of M-20 and PP after debonding, resulting from different PTs. Nanofibers of PP were observed on both surfaces for samples that underwent interfacial fracture (Figure 7a–c). For samples that displayed in-plane fracture, the debonded surfaces display nearly identical porous structure associated with the 20 nm membrane (Figure 7d). Table 2 summarizes the analysis of PP fibers on the debonded surfaces of the 20 nm PES/PP specimens after different PTs, for samples displaying an interfacial fracture. Correspondingly, *G*_c_ values calculated based on eq 3 are also summarized in Table 2. Note that for samples fractured via membrane failure (e.g., the case of Ar/O_2_ in Figure 7d), the details of the interlocking nanofibers cannot be imaged.

**Figure 7:**
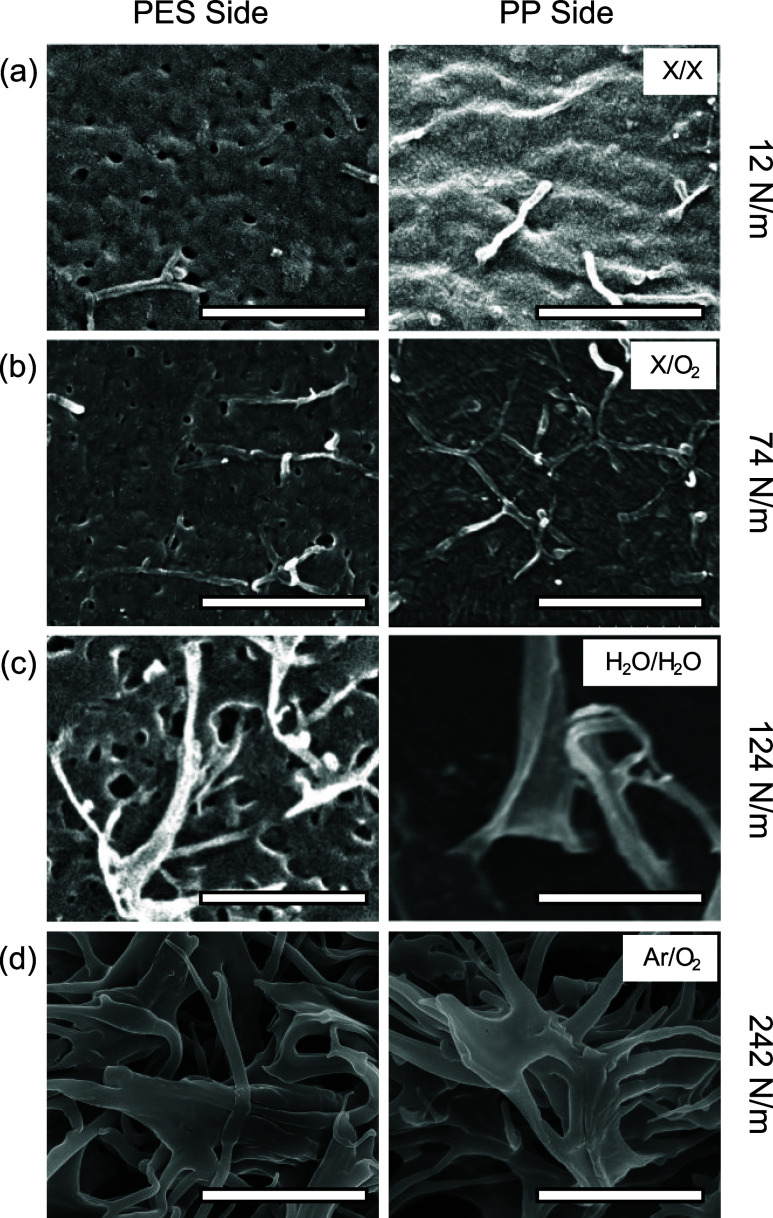
Debonded surfaces of M-20 membrane and corresponding PP for the following PT: (a) X/X, (b) X/O_2_, (c) H_2_O/H_2_O, and (d) Ar/O_2_. The experimental *G*_c_ values for the corresponding PT are noted to the right of the respective images; the scale bar for (a–c) is 500 nm and for (d) is 5 μm.

As shown in Table 2, the calculated *G*_c_ values based on the PP nanofibers were significantly lower than the experimental values (Figure 6), especially for plasma-treated samples. The results suggest that for the 20 nm PES membranes (small pore size and low surface porosity), the improved chemical interaction by PT can significantly raise the critical stress for crack initiation. As shown in Figure 6 and Table 2, PT raised the *G*_c_ values by orders of magnitude (from <1 N/m in X/X to >10 N/m for several PT combinations). Such enhancement may not be as significant in the case of 200 nm, where the mechanical interlocking is dominant (larger pores, higher surface porosity). However, for 20 nm PES membranes, especially M-20, the increase in chemical interactions (as seen from FTIR scans in Figure S3) can effectively increase the threshold of crack initiation, which translates to a more significant increase in *G*_c_. It is worth emphasizing that the mechanical interlocking by the PP nanofibers in these 20 nm membranes, despite providing less volumetric energy than the 200 nm membranes, is still necessary for achieving large *G*_c_ values. Such synergistic improvement in adhesion energy by interlocking and chemical bonding is commonly observed.^54^

## Discussion

Adhering polymers to nonreactive substrates is a challenging task due to their low surface energies. Plasma treatment has been used historically to increase the surface energy of these materials by the incorporation of polar functional groups, depending on the plasma content and the polymer being treated. PT also causes other chemical changes, such as carbon–carbon scission (leading to saturated and low-molecular-weight oxidized materials) and hydrogen abstraction (leading to cross-linking), which can also enhance adhesion.^55^ Improvement in adhesion, as measured by 90° peel tests, on metal films deposited on Kapton,^56^ polyimide,^57^ BPDA-PDA,^58^ PET, and PTFE^59^ has been observed for reactive neutral/ions/electron/photons individually, and for a mixture of all of the aforementioned species. Air plasma treatment at low pressure has been known to cause about 220% improvement in peel forces (from 250 N/m to 800 N/m) for 1.5 μm-thick SiO_*x*_ coatings on PC.^29^ The oxidative etching due to plasma is accelerated for oxygen-containing polymers such as PMMA, PET, and PC as compared to just hydrocarbon polymers such as PE and PP.^29^ Lap-shear bonding enhancement between two PP substrates joined by epoxy has shown about a 10-fold increase in the bond strength upon plasma treatment.^60,61^ Similarly, adhesion between PP to epoxy was improved by a factor of 5–7, and PP to aluminum by a factor of 2.5 due to low-pressure air plasma treatment.^62,63^ A comparison of the shear strength of the bond between PP and epoxy using a lap-shear test showed that air plasma caused an enhancement of 53–211%, whereas oxygen plasma caused an enhancement of 127–387%.^38^

In comparison to these nonporous substrates, no reports can be found on the impact of plasma treatment on the adhesion of porous membranes. In this study, we showed that the PT can improve the adhesion strength between PP and PES membranes. Depending on the combinations of plasma treatments, the interfacial fracture toughness values increase by 150–1700% and 1300–3700% for 20 and 200 nm membranes, respectively. Some of the samples display stronger adhesion than the strength of the membranes, which leads to fracture of the membranes either in-plane or through-thickness. Comparing the upper range of *G*_c_ values observed for PP/PES membranes (350 N/m) and PP/dense PES films (20 N/m), it is evident that mechanical interlocking caused by pore infiltration plays a dominant role in membrane adhesion. The data presented here showed that plasma treatments alone raised the *G*_c_ between PES film and PP film from nonmeasurable (thermodynamic work of adhesion between PES and PP is around 0.026 N/m) to 5–20 N/m, a 2–3 orders of magnitude enhancement. In comparison, mechanical interlock alone increases the *G*_c_ from nonmeasurable (between untreated PES film and PP film) to 110–160 N/m for 200 nm membranes and to 10–100 N/m for 20 nm membranes, a 3–4 orders of magnitude enhancement.

Chemical bonding and mechanical interlocking affect adhesion through the interface and bulk adhesive material, respectively. The synergy between them is reflected in two aspects. On one hand, mechanical interlocking increases the contact area and the adhesion contributed by chemical interaction. On the other hand, stronger chemical interaction strengthens the anchoring effect of mechanical interlocking and hence the energy dissipation in the bulk adhesive material. The two effects are not simply summative, as studies have shown that the enhancement in chemical bonding can lead to increased bulk viscoelastic dissipation during debonding.^64^ Most likely, the increased chemical interactions in the nonporous PP/PES interfaces increase the stress required for crack initiation and growth in those regions. The significantly enhanced *G*_*c*_ values for the membranes reported here are the result of the synergistic effect of both the enhanced chemical interactions between the PP and PES and the effective mechanical interlocking caused by the pore intrusion.

## Conclusion

This study investigated the use of plasma treatment to enhance the adhesion between porous PES membranes and supporting PP substrates under industrially relevant thermomechanical bonding conditions. Different combinations of plasma treatment effectively increased the surface energy of dense PES films and PP, which led to significant improvements in *G*_c_ values for adhesion between them. For both 200 and 20 nm PES membranes, improvements in *G*_c_ values were observed for the majority of the PT combinations. For 200 nm membranes, PT resulted in 10–220% enhancement in *G*_c_ values, and no major differences were observed between the two membrane chemistries (U-200 vs M-200). For 20 nm membranes, plasma treatments appear to have different impacts on U-20 and M-20. For U-20 membranes, large enhancements (200 and 300%) in *G*_c_ were only observed for Ar/O_2_ and O_2_/O_2_ combinations, respectively. For M-20 membranes, all plasma treatments resulted in enhancement of *G*_c_, with up to 2000% for the Ar/O_2_ combination. For the majority of the systems examined, interfacial debonding remained the dominant mechanism. However, for systems that displayed large *G*_*c*_ values, membrane fractures were observed when the adhesion strength exceeded the membrane strength during debonding. The study shows that plasma treatment is an effective method to improve the adhesion strength for membrane devices under high-throughput thermomechanical bonding conditions.

## Supplementary Material



## Notes

Data for surface energy components of water and diiodomethane (Table S1); contact angles of DIW and DIM on PP and PES (Figure S1); XPS spectra of PP with different PT (Figure S2); FTIR spectra of PES with different PT (Figure S3); AFM topographical scans of PP with different PT (Figure S4); surface energy, and uniaxial tensile response of M-20 after different PT (Figure S5); AFM topographical scans of 20 nm PES membranes after different PT (Figure S6); AFM topographical scans of 200 nm PES membranes after different PT (Figure S7); membrane surfaces of 20 nm PES membranes after Ar and H_2_O PT (Figure S8); enhancement of *G*_*c*_ in 200 nm PES membranes with respect to dense PES films (Figure S9); and enhancement of *G*_c_ in 20 nm PES membranes with respect to dense PES films (Figure S10) (PDF)

## List of Symbols

*d*_f_: fiber diameter
*E*: elastic modulus
*f*_c_: degree of crystallinity
*G*_c_: interfacial fracture toughness
*G*_c,est_: estimated interfacial fracture toughness
*L*_0_: polymer infiltration depth
*M*_n_: number-average molecular weight
*T*: tensile toughness
*T*_g_: glass transition temperature
*T*_m_: melting temperature
η_0_: steady-state viscosity
Φ: overall porosity of membrane
Φ_s_: surface porosity of membrane
γ^d^/*γ* ^p^: dispersive or polar component of surface energy
ρ_f_: density of fibers per unit area
σ_y_: yield strength
θ: contact angle

## List of Abbreviations

AFM: atomic force microscopy
BPDA-PDA: poly­(*p*-phenylene benzophenone tetracarboximide)
DIW: deionized water
DIM: diiodomethane
FTIR: Fourier transform infrared spectroscopy
M-20/M-200: acrylamide-modified 20 or 200 nm poly­(ethersulfone) membrane
MF: microfiltration
PC: polycarbonate
PDI: polydispersity index
PDMS: poly­(dimethylsiloxane)
PE: poly­(ethylene)
PEKK: poly ether-ketone-ketone
PES: poly­(ethersulfone)
PET: poly­(ethylene terephthalate)
PMMA: poly­(methyl methacrylate)
PP: poly­(propylene)
PT: plasma treatment
PTFE: poly­(tetrafluoroethylene)
PVDF: poly­(vinylidene difluoride)
SEM: scanning electron microscopy
T-20/T-200: acrylate-modified 20 or 200 nm poly­(ethersulfone) membrane
U-20/U-200: unmodified 20 or 200 nm poly­(ethersulfone) membrane
UF: ultrafiltration
XPS: X-ray photoelectron spectroscopy
