# Supplementary material for: Enhancing Membrane Adhesion to Polymeric Substrates via Plasma Treatment
Source: ACS Appl Polym Mater. 2026 Mar 16;8(6):3947–57. doi: 10.1021/acsapm.5c04051 (PMC13036713; doi:10.1021/acsapm.5c04051)
Supplement: Supplementary file 1 [file ap5c04051_si_001.pdf]

Enhancing Membrane Adhesion to Polymeric Substrates via Plasma Treatment

Rajan Jain[1*], Christina Carbrello[2], Kathy Youngbear[2], Sean Foley[2], Rong Long[1], Yifu Ding[1*]

[1]Membrane Applications, Science, and Technology (MAST) Center,
Paul M. Rady Department of Mechanical Engineering,
University of Colorado Boulder, Boulder, CO 80309, USA

[2]MilliporeSigma, 80 Ashby Rd, Bedford, MA 01730, USA

**All correspondence should be addressed to rajan.jain@colorado.edu or yifu.ding@colorado.edu*

Supporting Tables

Table S1. Surface energy components of water and diiodomethane.

Liquid	γ_{lg}^{d} (mN/m)	γ_{lg}^{p} (mN/m)	γ_{lg} (mN/m)
Water	21.8	51	72.8
Diiodomethane	50.8	0	50.8

Supporting Figures

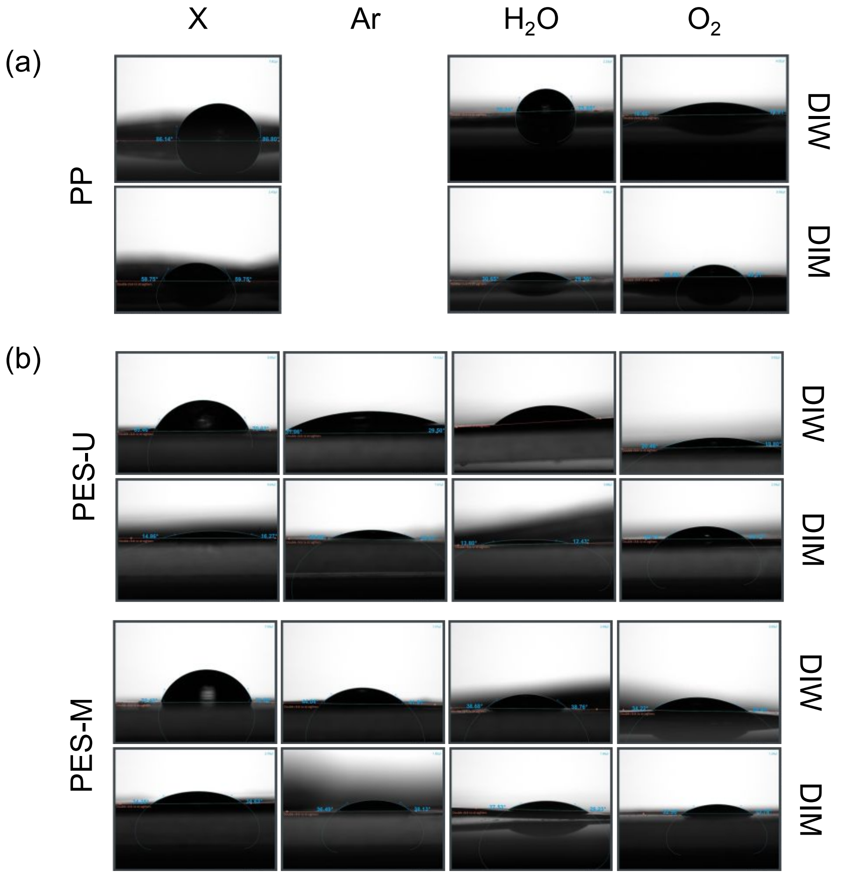

Figure S1. Contact angles of deionized water (DIW) and diiodomethane (DIM) on (a) PP and (b) PES-U and PES-M.

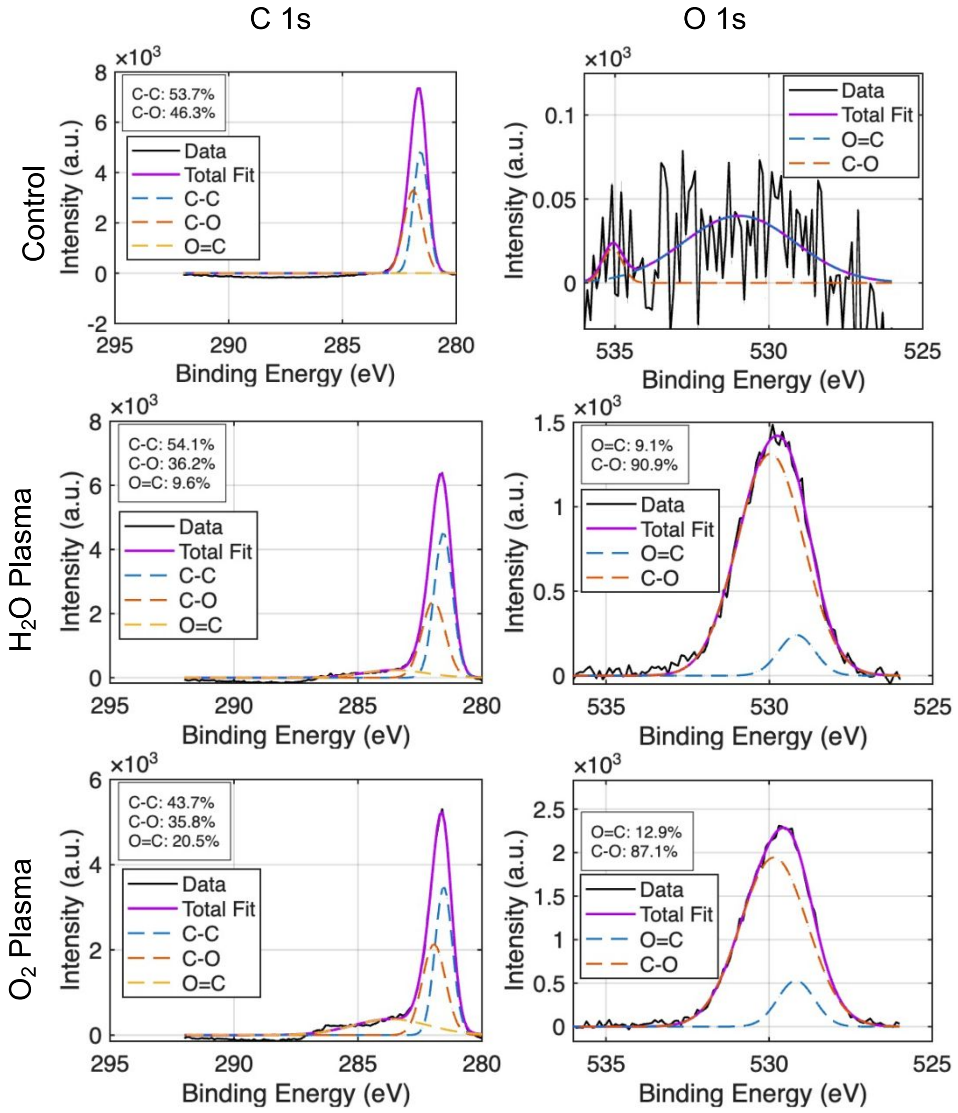

Figure S2. C1s and O1s spectra of PP with H_2O and O_2 PT.

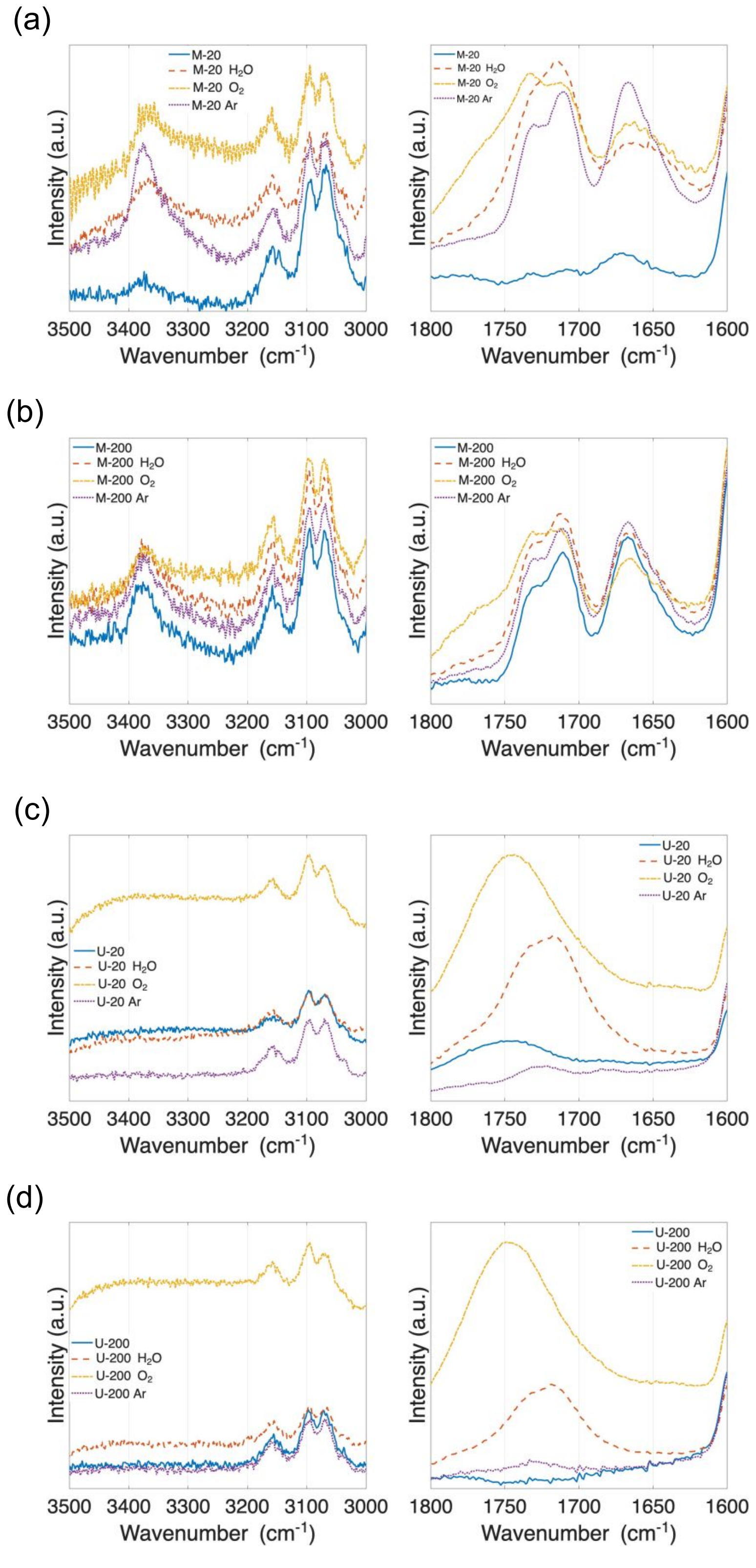

Figure S3. FTIR spectra in 1600-1800 cm^{-1} and 3000-3500 cm^{-1} for (a) M-20, (b) M-200, (c) U-20, and (d) U-200.

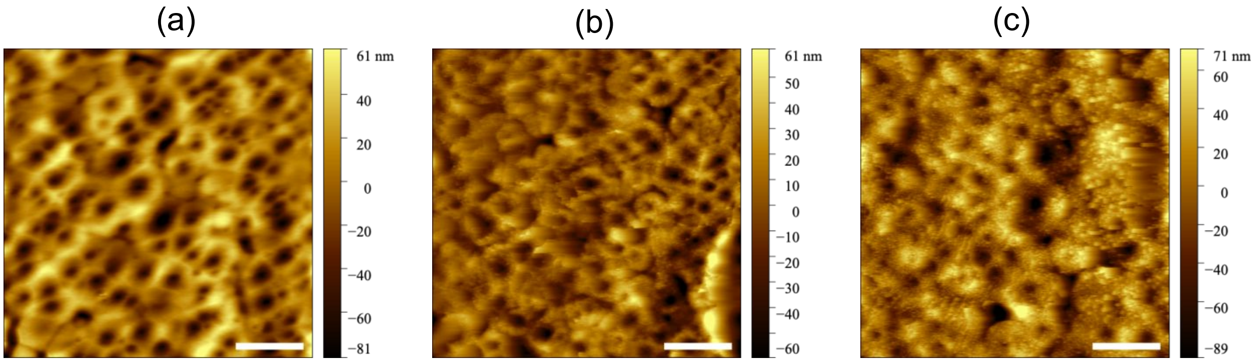

Figure S4. AFM topographical scans (25×25 μm^2) of PP treated with (a) no plasma, (b) H_2O plasma, and (c) O_2 plasma.

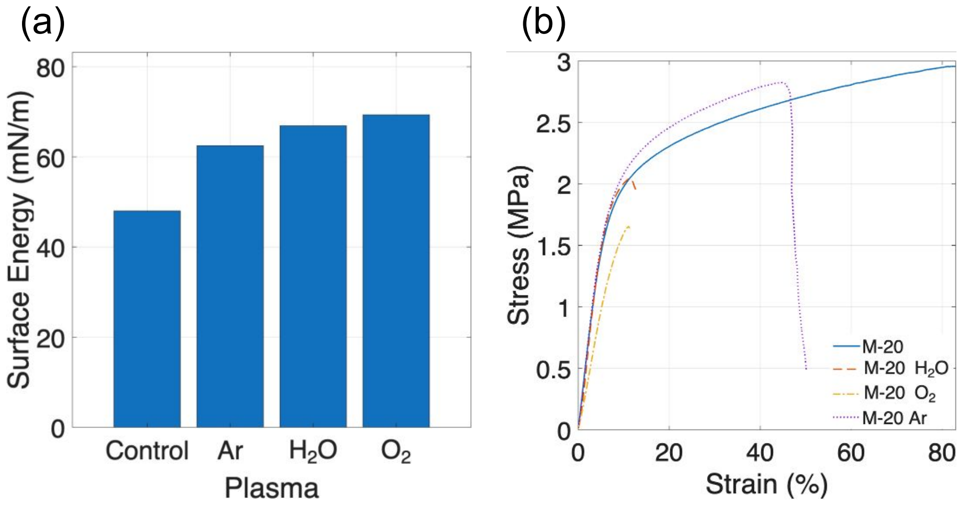

Figure S5. Properties of M-20 after different plasma treatments: (a) surface energy of densified films and (b) uniaxial stress-strain response.

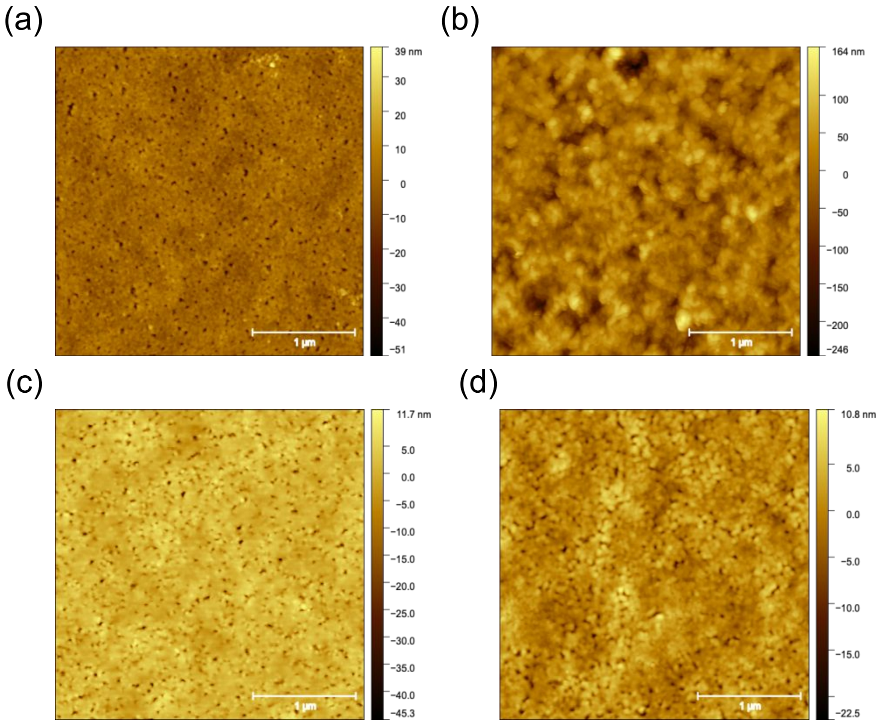

Figure S6. AFM topographical scans (3×3 μm^2) of (a) U-20, (b) O_2 plasma treated U-20, (c) M-20, (d) O_2 plasma treated M-20 membranes.

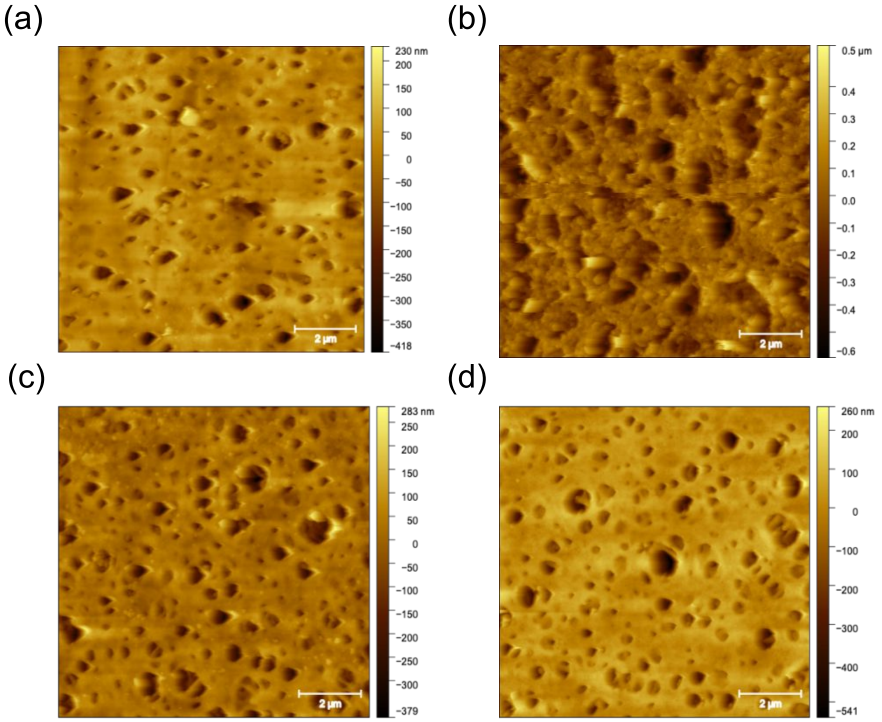

Figure S7. AFM topographical scans (10×10 μm^2) of (a) U-200, (b) O_2 plasma treated U-200, (c) M-200, (d) O_2 plasma treated M-200 membranes.

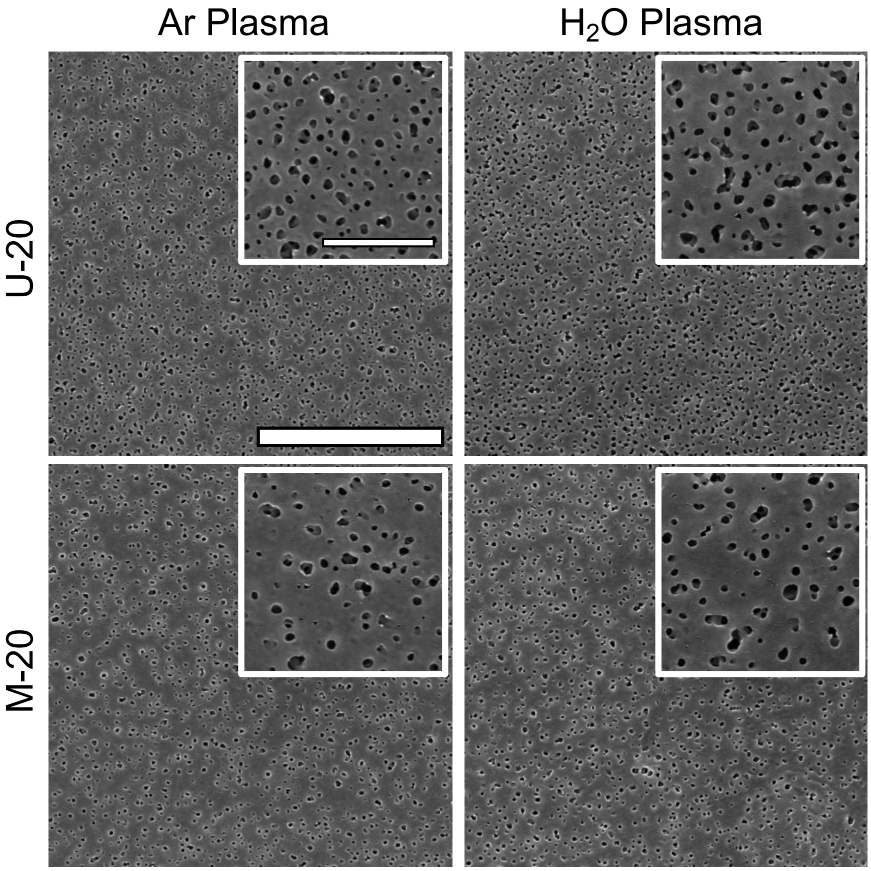

Figure S8. Membrane surfaces of U-20 and M-20 with Ar and H_2O PT (scale bar is 5 μm and inset scale bar is 500 nm).

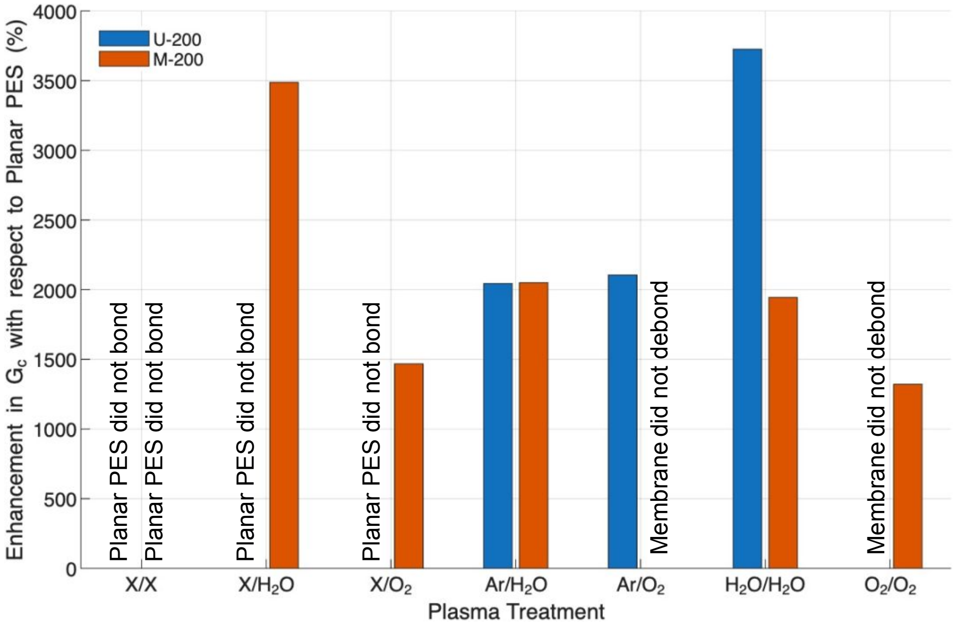

Figure S9. Enhancement in G_c in porous 200 nm PES membranes with respect to dense PES films.

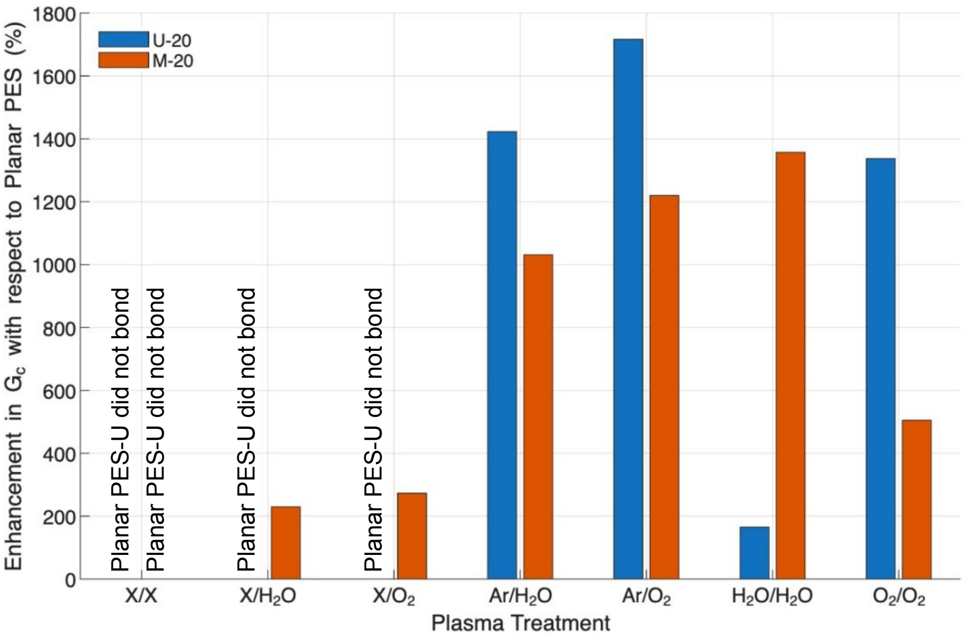

Figure S10. Enhancement in G_c in porous 20 nm PES membranes with respect to dense PES films.
